# Commercial Antivenoms Exert Broad Paraspecific Immunological Binding and In Vitro Inhibition of Medically Important *Bothrops* Pit Viper Venoms

**DOI:** 10.3390/toxins15010001

**Published:** 2022-12-20

**Authors:** Jaffer Alsolaiss, Nessrin Alomran, Laura Hawkins, Nicholas R. Casewell

**Affiliations:** 1 Centre for Snakebite Research & Interventions, Liverpool School of Tropical Medicine, Liverpool L3 5QA, UK; 2 Tropical Disease Biology Department, Liverpool School of Tropical Medicine, Liverpool L3 5QA, UK

**Keywords:** snakebite, antivenom, neglected tropical disease, venom toxins, paraspecificity

## Abstract

Snakebite envenoming is a life threatening neglected tropical disease that represents a considerable public health concern in the tropics. Viperid snakes of the genus *Bothrops* are among those of greatest medical importance in Latin America, and they frequently cause severe systemic haemotoxicity and local tissue destructive effects in human victims. Although snakebite antivenoms can be effective therapeutics, their efficacy is undermined by venom toxin variation among snake species. In this study we investigated the extent of paraspecific venom cross-reactivity exhibited by three distinct anti-*Bothrops* antivenoms (Soro antibotrópico-crotálico, BothroFav and PoliVal-ICP) against seven different *Bothrops* pit viper venoms from across Latin America. We applied a range of in vitro assays to assess the immunological binding and recognition of venom toxins by the antivenoms and their inhibitory activities against specific venom functionalities. Our findings demonstrated that, despite some variations, the monovalent antivenom BothroFav and the polyvalent antivenoms Soro antibotrópico-crotálico and PoliVap-ICP exhibited extensive immunological recognition of the distinct toxins found in the different *Bothrops* venoms, with Soro antibotrópico-crotálico generally outperformed by the other two products. In vitro functional assays revealed outcomes largely consistent with the immunological binding data, with PoliVap-ICP and BothroFav exhibiting the greatest inhibitory potencies against procoagulant and fibrinogen-depleting venom activities, though Soro antibotrópico-crotálico exhibited potent inhibition of venom metalloproteinase activities. Overall, our findings demonstrate broad levels of antivenom paraspecificity, with in vitro immunological binding and functional inhibition often highly comparable between venoms used to manufacture the antivenoms and those from related species, even in the case of the monovalent antivenom BothroFav. Our findings suggest that the current clinical utility of these antivenoms could possibly be expanded to other parts of Latin America that currently suffer from a lack of specific snakebite therapies.

## 1. Introduction

Snakebite envenoming is a life-threatening, morbidity-causing, World Health Organization (WHO)-listed neglected tropical disease [1]. The populations at greatest risk of snakebite occupy the tropical and sub-tropical regions of the world, and the burden of disease is greatest in rural areas of South and Southeast Asia, sub-Saharan Africa, and Latin America [2]. Collectively, it is estimated that perhaps as many as 1.8 million people are envenomed annually, of whom 90,000–138,000 die, and many more suffer life-long morbidity as the result of associated physical and mental trauma [1,2]. In Latin America, it is estimated that between 80,000 and 129,000 individuals are envenomed annually, though the true incidence may be greater due to extensive global underreporting of snakebite [3].

Pit vipers of the genus *Bothrops* (Viperidae: Crotalinae), often referred to as lanceheads or lancehead vipers, are of greatest medical importance in Central and South America, as they are responsible for causing the majority of cases of severe envenoming [1,3,4]. Snakes of this genus inhabit a large geographical distribution, ranging from southern parts of Mexico in the north, to Argentina in the south, and also include Caribbean island populations, such as *Bothrops lanceolatus* on Martinique and *B. caribbaeus* on St. Lucia. The pathophysiology of envenomings by *Bothrops* spp. can be variable, but victims typically present with haemorrhage, hypotension, impaired blood coagulation, inflammation, acute renal damage, and/or local tissue damage, the latter of which can result in irreversible sequelae requiring surgical interventions such as the removal of necrotic flesh or amputation of extremities or digits [3,5,6].

Venom proteomic [7,8] and venom gland transcriptomic [9,10] studies have provided considerable insights into the venom composition, and thus the aetiological toxins responsible for causing envenoming pathology, of a large number of *Bothrops* species over many years. In line with many viperid species, toxins of the snake venom metalloproteinase (SVMP), snake venom serine proteinase (SVSP) and phospholipases A_2_ (PLA_2_) gene families are typically the most abundant components found in *Bothrops* venom [11]. Although considerable inter-specific variation in venom composition exists within the genus, the SVMPs are typically the mostly abundant of these [11] and this toxin family can represent as much as 75% of the total proteins present in venom, as observed in *B. lanceolatus* [12]. SVMPs are enzymes with multiple functions that participate in both local and systemic effects of snakebite envenoming, mostly notably via the cleavage of basement membrane proteins of blood vessels resulting in haemorrhage, or via the activation or degradation of blood clotting factors resulting in coagulopathy, though these toxins can also interact with platelets and inflammatory mediators [13]. The SVSP toxin family also contribute to venom induced coagulopathy, with many representatives classified as thrombin-like enzymes due to their fibrinogenolytic mode of action [14], while PLA_2_s are more typically implicated in contributing to the severity of local envenoming effects, including myotoxicity, though they can exert anticoagulant activities via inhibition of platelet aggregation [15,16]. Abundances of these toxin types also vary across the genus, with PLA_2_s reaching a maximum abundance of 45% in *B. asper*, while SVSPs peak at 29% in southern populations of *B. jararaca* [11,17,18]. Such inter-specific toxin family variations also extend to other minor venom components, including C-type lectins and disintegrins, while intra-specific sex- and population-based, along with ontogenetic, venom variation has also previously been described from this genus of snakes [18,19,20,21].

The only specific treatments for snakebite envenoming are antivenoms, which consist of polyclonal antibodies sourced from venom hyper-immunised animal plasma/sera [1], and their importance is highlighted by inclusion on the list of essential medicines published by the WHO (https://www.who.int/publications/i/item/WHO-MHP-HPS-EML-2021.02 accessed on 13 September 2022). There are two main types of antivenom, namely monospecific (or monovalent) antivenom, which is generated against the venom of a single snake species, and polyspecific (or polyvalent) antivenom, which is made using the venom of multiple snake species as immunogens. There are advantages and disadvantages to both approaches: in general, for many parts of the world polyspecific antivenom is more advantageous because of the presence of multiple biting species and a lack of effective diagnostic tools; thus a single therapy that can be used for a particular region is desirable. However, due to only a proportion of the antibodies present in polyspecific antivenom being specific to the snake species responsible for a bite, typically higher therapeutic doses have to be administered to effect cure [22], resulting in cost increases and a theoretical increased risk of adverse reactions. Moreover, despite the production of polyvalent antivenoms, venom variation renders many of these products with limited efficacy against different snake species not included in the immunising mixture, or even against different populations of the species whose venoms were used to generate the antivenom [23,24,25,26]. Sadly, this means that many people who suffer from snakebite envenoming do not have access to effective, specific therapeutics. The unpredictable nature of snake venom variation means that there is a strong rationale for testing the capability of existing antivenoms to inhibit the venom activities of snake species related to those used as immunogens. Evidence of paraspecific efficacy might increase the geographical utility of an antivenom, which could benefit patients by increasing access to effective treatment, while manufacturers may benefit by accessing new markets, so long as standards of safety and efficacy are upheld [27].

In this study, we explored the potential utility of three distinct antivenoms generated (at least partially) against medically important *Bothrops* spp. found in Central and South America at binding with and inhibiting the in vitro functional activities of seven distinct *Bothrops* venoms spanning a broad geographical distribution ranging from Costa Rica to Brazil (Table 1). The antivenoms included the polyvalent anti-*Bothrops* Brazilian product Soro antibotrópico-crotálico, the trivalent anti-*Bothrops asper* (and other pit vipers) antivenom PoliVal-ICP from Costa Rica, and the *B. lanceolatus*-specific monovalent antivenom BothroFav (Table 2). Our findings, sourced from a variety of in vitro assays, demonstrated that these three distinct products exhibit variable binding and neutralisation potencies against the seven venoms under study, though in the majority of experiments each antivenom showed clear evidence of venom toxin recognition and at least some inhibition of functional activities. Collectively our findings suggest that the PoliVap-ICP and BothroFav antivenoms show particular promise for wider therapeutic use in regions where the current provision of specific antivenom is limited. The data presented here strongly advocate for future studies to preclinically validate our findings of paraspecific efficacy to facilitate downstream clinical use of these antivenoms in snakebite victims.

## 2. Results

### 2.1. Visualisation and Quantification of Venom-Antivenom Binding Interactions

**Figure 1:**
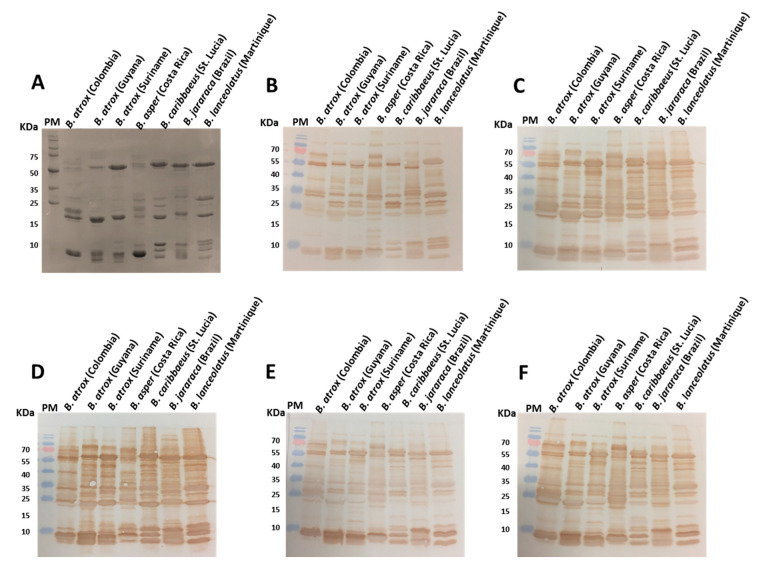
SDS-PAGE gel electrophoretic profiles of *Bothrops* venoms and their immunological recognition by commercial antivenoms. (**A**) Venom samples were separated by reduced SDS-PAGE gel electrophoresis and visualised by Coomassie blue staining. The same venom samples were also transferred to nitrocellulose membranes and incubated with 1:5,000 dilutions of primary antibodies: (**B**) Soro anticrotálico, (**C**) Soro antibotrópico-crotálico, (**D**) Bothrofav, (**E**) PoliVap-ICP (2006), (**F**) PoliVap-ICP (2013). PM indicates protein marker. Note that the molecular mass standards differ between the SDS-PAGE gel and the immunoblots.

To visualise the immunological recognition of the commercial antivenoms against the various venom proteins found in the seven *Bothrops* venoms, we subjected each venom to reduced SDS-PAGE gel electrophoresis and Western blotting. As anticipated, the reduced SDS-PAGE profiles illustrated considerable inter-specific variation in the molecular masses and relative abundances of the different proteins found in the various venoms (Figure 1A). The venoms displayed a wide range of different proteins, predominately from ~10-60 kDa in mass, though the majority of the high-intensity bands, indicative of abundant venom proteins, were particularly noticeable at <25 kDa or ~60 kDa. Despite evidence of venom variation, immunoblotting experiments with each of the commercial antivenoms revealed extensive immunological recognition of the proteins found in each of the snake venoms (Figure 1B–F). This broad cross-species recognition also extended to the anti-*Crotalus* Soro anticrotálico antivenom, used as a non-*Bothrops* control, though the intensities obtained were lower than those observed with the various anti-*Bothrops* antivenoms, except for the older batch of PoliVap-ICP. Surprisingly, there were very little differences in immunological recognition and binding intensities between the other antivenoms, with both the monovalent BothroFav (specific to *B. lanceolatus*) and the trivalent PoliVap-ICP (2013, *Bothrops*-specific to *B. asper*) exhibiting broad recognition across all venoms, and in a highly comparable manner to the polyvalent Soro antibotrópico-crotálico antivenom (Figure 1C–F).

**Figure 2:**
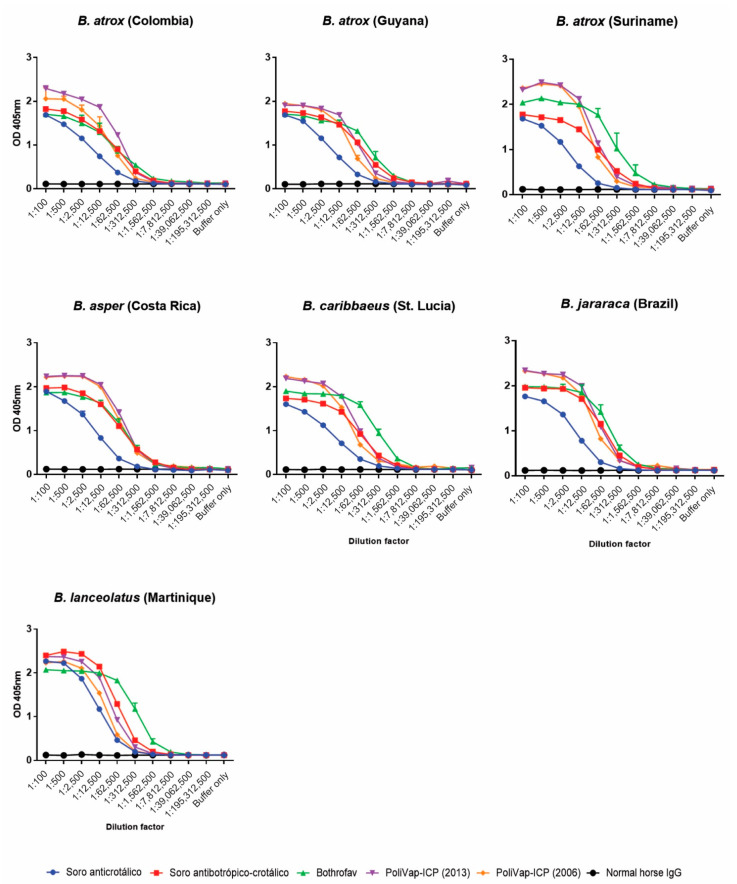
End-point titration ELISA analyses showing quantification of immunological binding between the commercial antivenoms and each of the *Bothrops* venoms. The various antivenoms (Soro anticrotálico, Soro antibotrópico-crotálico, BothroFav, PoliVap-ICP 2006, PoliVap-ICP 2013) and normal horse IgG (negative control) were tested at an initial dilution of 1:100 and serially diluted fivefold. Error bars (where visible) represent standard deviation (SD) of duplicate measurements.

To quantify the amount of immunological binding between the various commercial antivenoms and *Bothrops* venoms, we used end-point titration ELISAs. The resulting binding profiles of titrated antivenoms revealed a consistent pattern whereby the non-*Bothrops* control antivenom (Soro anticrotálico) exhibited lowest binding across all seven venoms, as anticipated (Figure 2). However, in contrast with the Western blotting experiments, quantifications of immunological binding revealed some, generally minor, differences between the antivenoms. For example, the BothroFav antivenom exhibited higher binding levels to *B. lanceolatus*, *B. caribbaeus* and Suriname *B. atrox* venom, while PoliVap-ICP exhibited greatest binding against *B. asper* and Colombian *B. atrox* venoms (Figure 2). The Soro antibotrópico-crotálico antivenom failed to provide highest binding levels against any of the venoms, though it was highly comparable to BothroFav and PoliVap-ICP against both *B. jararaca* and *B. atrox* (Guyana) venom. Little difference was observed between the binding profiles of the two batches of the PoliVap-ICP antivenom tested, perhaps except against venom from Colombian *B. atrox* (Figure 2).

**Figure 3:**
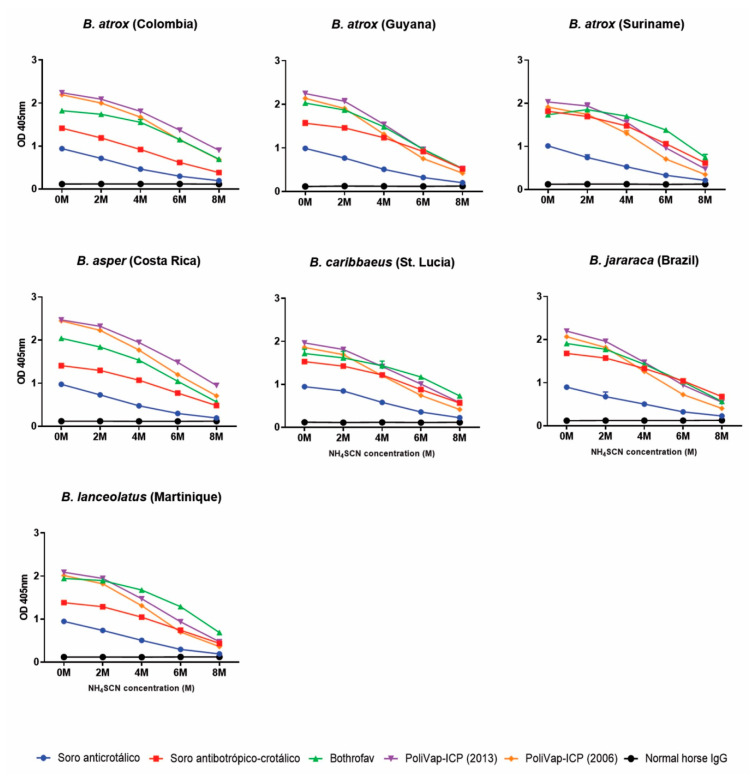
Avidity ELISA analyses showing quantification of the strength of binding between the commercial antivenoms and each of the *Bothrops* venoms. Avidity ELISA analyses showing immunological binding of the commercial antivenoms against each of the *Bothrops* venoms in the presence of increasing molarities of the chaotropic agent ammonium thiocyanate (NH_4_SCN). The various antivenoms (Soro anticrotálico, Soro antibotrópico-crotálico, BothroFav, PoliVap-ICP 2006, PoliVap-ICP 2013) and normal horse IgG (negative control) were tested at a single dilution of 1:10,000 in the presence of increasing concentrations of ammonium thiocyanate (0–8 M). Error bars (where visible) represent SD of duplicate measurements.

To determine the strength of the binding interactions between each of the commercial antivenoms and the various *Bothrops* venoms we performed avidity ELISAs, which quantify binding levels in the presence of increasing concentrations of a chaotropic agent (ammonium thiocyanate, NH_4_SCN; 0–8 M) that disrupts protein binding. The results revealed that the anti-*Bothrops* antivenoms generally exhibited high binding levels against the various *Bothrops* venoms, often in a comparable manner against the different species tested, in the presence of up to 4 M ammonium thiocyanate (Figure 3). The binding profiles were largely consistent with the end-point ELISA, with BothroFav exhibiting highest avidity against *B. lanceolatus* venom and, to a lesser extent, also against *B. caribbaeus* and *B. atrox* Suriname, while PoliVap-ICP antivenom performed best against *B. asper* and Colombian *B. atrox* venom (Figure 3). The Soro antibotrópico-crotálico antivenom was generally outperformed by BothroFav and PoliVap-ICP in this assay, though for at least three *Bothrops* species (i.e., *B. jararaca*, *B. caribbaeus* and *B. atrox* Suriname) it provided nearly comparable avidity binding profiles, and was clearly superior to the non-*Bothrops* control antivenom Soro anticrotálico (Figure 3).

### 2.2. In Vitro Venom Neutralisation by Commercial Antivenoms

**Figure 4:**
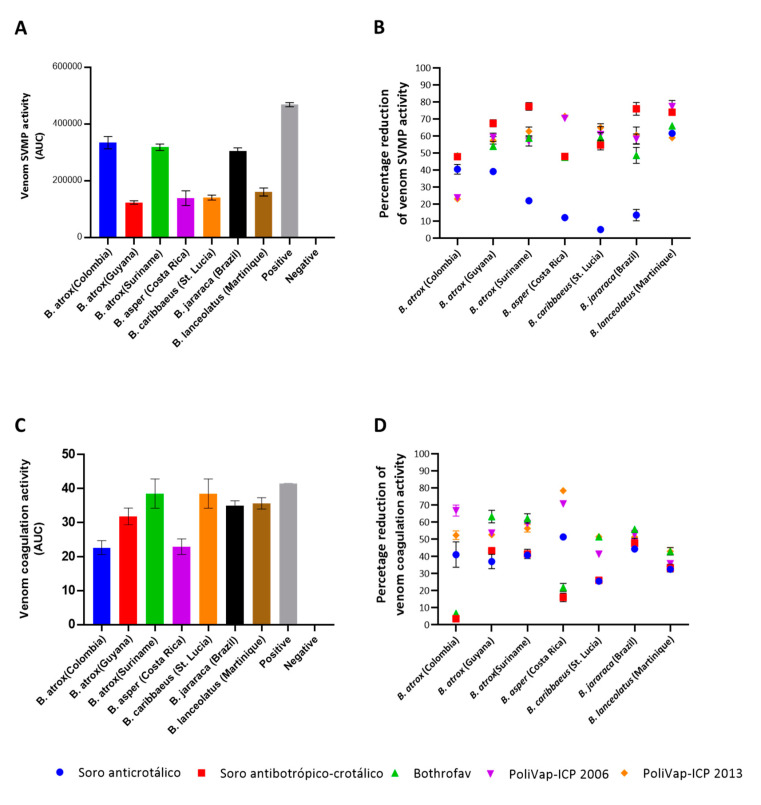
The in vitro SVMP and coagulopathic venom activities of *Bothrops* venoms and the inhibitory capability of the commercial antivenoms. (**A**) The enzymatic snake venom metalloproteinase (SVMP) activity of each venom displayed as the area under the curve (AUC) of kinetic profiles resulting from the cleavage of a quenched fluorogenic substrate over time. The positive control was *Echis ocellatus* venom and the negative control was plasma with the addition of PBS instead of venom. (**B**) Inhibition of venom SVMP activities by the various commercial antivenoms displayed as the percentage reduction of venom only activities displayed in (**A**). (**C**) The coagulopathic activity of each venom displayed as the AUC resulting from increases in absorbance stimulated by venom-induced clotting over time. (**D**) Inhibition of coagulopathic venom activities by the various commercial antivenoms displayed as the percentage reduction of venom only activities displayed in (**C**). For all data shown, data points represent means of triplicate readings, and error bars represent standard errors of the means (SEMs). See also Figures S1 and S2.

To assess the capability of the commercial antivenoms to neutralise the functional activities of *Bothrops* venoms, we first employed two different in vitro assays, which specifically related to SVMP toxin and coagulopathic venom activities. The enzymatic SVMP activities of the venoms used in this study were kinetically measured via cleavage of a specific quenched fluorogenic substrate (ES010, R&D Biosystems) in the presence or absence of the various antivenoms. As anticipated based on prior knowledge of *Bothrops* spp. venom composition [11], all seven venoms exhibited detectable SVMP activity, though the venoms of *B. atrox* (Colombia and Suriname) and *B. jararaca* exhibited activities noticeably higher than those of the other four venoms (Figure 4A). Experiments assessing SVMP neutralisation revealed that all the commercial antivenoms had a potent inhibitory effect on the SVMP activities of the seven venoms at the concentration tested, except for the control, non-*Bothrops* antivenom, Soro anticrotálico (Figure 4B and Figure S1). Indeed, the mean reduction of SVMP activities across the various venoms exceeded 50% for all anti-*Bothrops* antivenoms (63.70% for Soro antibotrópico-crotálico; 58.14% and 57.08% for the two PolyVap-ICP batches; 54.68% for BothroFav), with Soro antibotrópico-crotálico consistently exhibiting the highest inhibitory effects (range 47.94–77.47% inhibition). Although antivenom inhibitory capabilities were largely comparable against several venoms (e.g., *B. atrox* Guyana, *B. caribbaeus* and *B. lanceolatus*), PolyVap-ICP exhibited superior potency against *B. asper*-induced SVMP activities, while Soro antibotrópico-crotálico performed best against *B. jararaca* and Suriname *B. atrox* (Figure 4B). Interestingly, none of the antivenoms performed well against Colombian *B. atrox* venom, with inhibitory reductions ranging from ~23–48%. Perhaps surprisingly, the control Soro anticrotálico antivenom exhibited comparable inhibitory effects to the other antivenoms against *B. lanceolatus* and Colombian *B. atrox* venom, but was largely ineffective against the SVMP activity of the remaining venoms.

To assess the coagulopathic activity of the *Bothrops* venoms, we used a previously described absorbance-based plasma clotting assay [28,29]. All seven *Bothrops* venoms exhibited procoagulant activities at the dose tested (1 µg), and thus stimulated clot formation quicker than that of the plasma only control (Figure 4C). Interspecific differences in venom activity were more modest than observed in the SVMP assay, with many venoms exhibiting comparable coagulotoxic activities, though *B. atrox* Colombia and *B. asper* venoms exhibited lower potencies than the rest. Neutralisation experiments revealed a variable picture of functional inhibition, with mean antivenom-induced reductions of coagulotoxicity ranging from ~30–55% (30.27% for Soro antibotrópico-crotálico; 54.33% and 55.10% for the two PoliVap-ICP batches; 43.52% for BothroFav), though PoliVap-ICP provided the highest and broadest inhibition across the different venoms (range 35.90–78.42% [2006] and 43.03–78.42% [2013] inhibition) (Figure 4D and Figure S2). For some venoms (e.g., *B. jararaca* and *B. lanceolatus*) the various different antivenoms, including the control anti-*Crotalus* antivenom Soro anticrotálico, performed in a highly comparable inhibitory manner. However, the Soro antibotrópico-crotálico antivenom performed poorly against Colombian *B. atrox*, *B. asper* and *B. caribbaeus* venom, while BothroFav also underperformed against the first two of these venoms, though it did provide the greatest inhibitory effects against the remaining five venoms tested (all >40% inhibition) (Figure 4D).

**Figure 5:**
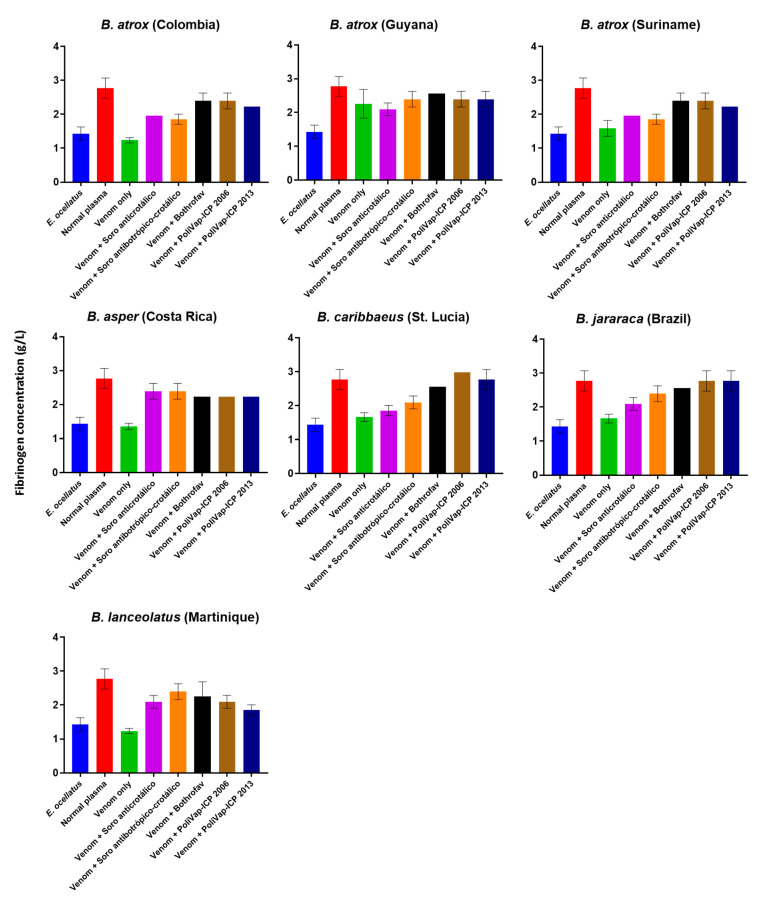
Commercial antivenoms reduce the consumption of fibrinogen stimulated by the various *Bothrops* venoms. Platelet poor plasma (PPP) was spiked with venom (0.6 ng) or venom and antivenom (0.5 µg) and ensuing fibrinogen concentrations calculated. Venom from *Echis ocellatus* was used as the positive control and unspiked PPP was used as the negative control. Error bars represent the standard deviation of duplicate measurements.

Next, we investigated the inhibitory effects of each of the commercial antivenoms against the various *Bothrops* venoms using a venom-spiking experiment with platelet-poor plasma (PPP) and measuring resulting fibrinogen concentrations. Fibrinogenolysis is an important component of venom-induced coagulopathy, and the depletion of fibrinogen is typically stimulated via the action of SVSP and/or SVMP toxin families acting either indirectly via upstream activation of clotting factors or directly via cleavage of fibrinogen [14]. Our findings revealed that all venoms stimulated fibrinogen consumption, with fibrinogen concentrations with the normal plasma control (2.77 g/L) substantially reduced by the various *Bothrops* venoms (1.29–1.96 g/L), except for *B. atrox* venom from Guyana, which was considerably less potent (2.26 g/L) (Figure 5). The resulting fibrinogen concentrations for the remaining six venoms were highly comparable to that induced by the positive control venom from *E. ocellatus* (1.43 g/L). The various antivenoms exhibited variable potencies in terms of protecting against fibrinogen consumption stimulated by the different venoms (Figure 5). Most of the antivenoms (including the Soro anticrotálico control) reduced the consumption of fibrinogen stimulated by each of the venoms, and in many instances to control levels. For some venoms, antivenom-induced protection was highly comparable amongst the different products (e.g., against *B. asper*, *B. atrox* Guyana and *B. lanceolatus*), while for other venoms clearer patterns of inhibitory potency emerged, including BothroFav and PoliVAP-ICP outperforming the Soro antibotrópico-crotálico antivenom against *B. caribbaeus* and the Colombian and Suriname populations of *B. atrox* (Figure 5).

## 3. Discussion

Pit vipers of the genus *Bothrops* cause most of the severe snakebites that occur in the Caribbean, Central America and South America [1,3]. Snakebite envenoming by *Bothrops* species manifests as potentially lethal systemic haemorrhage and coagulopathy, while extensive local tissue damage and inflammation occurs frequently and can lead to amputation and other irreversible local sequelae resulting in life-long morbidity [1,3]. The WHO guidelines specify that snakebites should be treated with antivenom, the only venom-specific therapeutics that exist today. However, this course of action is difficult to implement in disadvantaged regions with low antivenom availability, accessibility and/or affordability. Further, venom variation means that commercial antivenoms have limited geographical utility, and as such there are large parts of the tropical world for which no bespoke antivenom is manufactured, meaning that assessing the efficacy of existing products against snake species or populations for which no specific treatment exists (i.e., assessing their paraspecific efficacy) is of utmost importance [24,27]. Consequently, in this study we explored the extent to which three commercially available Central/South American antivenoms might have broad utility against *Bothrops* envenomings in the region.

Our study used seven different *Bothrops* venoms from five different species that covered a broad geographical distribution in Latin America and the Caribbean. In line with previous studies (summarised in [11,30]), the toxin compositions of these venom samples differed considerably, as observed by SDS-PAGE gel electrophoresis (Figure 1A). However, all venoms exhibited clear presence of detectable proteins at three major molecular mass regions (7–12 kDa, 18–30 kDa and 55–60 kDa), and these observations may therefore reflect inter-specific differences in abundances and isoform numbers within the major viper toxin families, such as the SVMP, PLA_2_ and SVSPs, rather than major differences in the presence or absence of these different toxin types. Despite this compositional variation, some functional activities of the venoms appeared highly consistent in our study. For example, all seven venoms substantially reduced fibrinogen concentrations in venom-spiked platelet poor plasma (Figure 5) and, while there were differences in potency, all were procoagulant to plasma (Figure 4C). These findings are largely consistent with previous reports on the coagulopathic activity of the *Bothrops* venoms under study [30,31,32], although Bourke et al. [32] additionally showed via thromboelastometric approaches that the fibrinogenolytic potency of *Bothrops* venoms can vary considerably. We did observe variation in SVMP venom activity, as measured by enzymatic assay, with the Suriname and Colombian populations of *B. atrox*, along with *B. jararaca* venom, exhibiting considerably higher activity than the remaining venoms (Figure 4A). These venom potency differences were not predicted based on the SDS-PAGE profiles and band intensity in the 55–60 kDa region (indicative of P-III SVMPs), or based on the literature, where *B. lanceolatus,* for example, has been reported to have the highest abundance of SVMPs in its venom (~75% [12]), but exhibited a more modest enzymatic activity that the three venoms mentioned above.

Despite these compositional and functional venom variations, the three anti-*Bothrops* antivenoms (Soro antibotrópico-crotálico, PoliVap-ICP and BothroFav) displayed extensive immunological recognition of these diverse toxin components, as clearly evidenced by both the Western blotting and ELISA experiments (Figure 1, Figure 2 and Figure 3). The Western blots were non-discriminatory, perhaps surprisingly revealing that the monovalent BothroFav antivenom recognised a comparable diversity of toxins found across the various *Bothrops* venoms to the trivalent (though *B. asper*-specific in terms of *Bothrops*) PoliVap-ICP and the polyvalent Soro antibotrópico-crotálico antivenoms (Figure 1). Endpoint titration and avidity ELISAs revealed some quantitative differences in antivenom-venom binding, with BothroFav generally exhibiting highest binding against the Caribbean and Suriname venoms from *B. lanceolatus*, *B. caribbaeus* and *B. atrox,* while PoliVap-ICP was superior against Central American *B. asper* and Colombian *B. atrox* venoms (Figure 2 and Figure 3). Interestingly, for each of these antivenoms we found evidence of paraspecific venom binding at comparable levels to that observed with the venom used for immunisation. While the Soro antibotrópico-crotálico antivenom was generally outperformed in these assays, most notably in the avidity ELISAs except against *B. jararaca* and *B. atrox* Suriname venoms, differences in immunological binding observed were relatively modest suggesting only a slightly reduced potency. Overall, these data provide strong support that each of these antivenoms contains antibodies capable of recognising and binding to a diverse range of toxins found in Latin American *Bothrops* venoms, irrespective of which venoms are used for the immunisation process.

Because levels of antibody-toxin binding are not necessarily reflective of toxin inhibition, we used three different in vitro functional assays to explore paraspecific venom neutralisation by the three antivenoms. The fluorogenic SVMP activity assay revealed data highly consistent with the immunological assays, with all three antivenoms performing in a largely comparable manner, though Soro antibotrópico-crotálico exhibited the highest mean percentage reductions in venom SVMP activity (Figure 4). Measures of venom-induced coagulopathy revealed a different inhibitory pattern, with Bothrofav and PoliVap-ICP exhibiting highly comparable neutralising potencies against procoagulant venom activities observed in the plasma assay, except that BothroFav was less effective against Colombian *B. atrox* and *B. asper* venom at the tested antivenom doses (Figure 4). Measures of fibrinogen concentrations in venom-spiked PPP revealed a similar pattern, with Bothrofav and PoliVap-ICP exhibiting highly comparable inhibitory profiles, and in this case including equipotency against both *B. atrox* and *B. asper* (Figure 5). Both antivenoms consistently outperformed Soro antibotrópico-crotálico in these two assays, suggesting that, despite extensive inhibition of enzymatic SVMP activity by this antivenom, perhaps other toxin types are contributing to the procoagulant venom phenotypes observed.

This work highlights the therapeutic potential of BothroFav, PoliVap-ICP and, perhaps to a lesser extent, Soro antibotrópico-crotálico antivenoms for broad use against the venom of medically important *Bothrops* species found across Central and South America and the Caribbean. Most notably, across the various immunological and functional in vitro assays employed herein, we found only minor differences in the binding and inhibitory capabilities of these three antivenoms, despite distinct immunogens being used to raise the polyclonal antibodies (Table 2). Each antivenom exhibited at least some capability to recognise, bind and inhibit the toxin activities of *Bothrops* snake venoms not used as immunogens, highlighting the potential paraspecific efficacy of such antivenoms, as also proposed elsewhere [31,33,34,35]. Overall, PoliVap-ICP performed well against most venoms tested in most assays, though perhaps surprisingly the monovalent product Bothrofav was highly comparable, apart from reductions in both binding and procoagulant toxin inhibition against Colombian *B. atrox* and *B. asper* venom. Except for the SVMP assay and for comparisons with the venom from *B. jararaca* used as an immunogen, the Soro antibotrópico-crotálico antivenom was generally outperformed by the other two products, though evidence of broad paraspecific cross-reactivity and toxin inhibition was observed. It is worth noting that in this study we were limited by using seven venoms sourced from five *Bothrops* species for our assessments of antivenom cross-reactivity. While these venoms span a broad geographical range (Table 1), there are many other *Bothrops* species (the genus contains >50 species), including medically important species such as *B. alternatus*, *B. moojeni* and *B. neuwiedi*, and since venom variation can be unpredictable it remains unclear whether patterns of broad paraspecific binding observed here would extend to other members of this speciose genus [24,25,36].

Although promising, these findings must next be validated using more robust models of antivenom efficacy, including small animal models of envenoming, to assess whether these antivenoms are capable of significantly reducing the lethal, coagulant, haemorrhagic and dermonecrotic activities of various *Bothrops* snake venoms in vivo [33,34,35,37]. These analyses could perhaps also be usefully complemented by proteomic-based analyses of antibody binding (i.e., ‘antivenomics’), to determine which toxins are depleted by antivenoms and which are not recognised due to venom variation [8,38,39]. Evidence of paraspecific preclinical efficacy and toxin depletion would provide a compelling basis to then explore the clinical efficacy of these antivenoms in observational studies of snakebite victims, particularly in regions of Latin America where species-specific antivenom therapies are currently not available. Expanding the clinical utility of existing antivenoms offers a potential short-term solution to reduce the severity of life-threatening systemic and morbidity-causing local envenoming caused by *Bothrops* pit vipers.

## 4. Material and Methods

### 4.1. Venom and Antivenom

Seven different *Bothrops* venoms from five different species were used in this study (Table 1). All lyophilised venoms were sourced from Kentucky Reptile Zoo (Salde, KY, USA), Latoxan (France), or the historical venom collection at the Centre for Snakebite Research & Interventions (CSRI), Liverpool School of Tropical Medicine (UK). Lyophilised venoms were stored at 4 °C and reconstituted with phosphate-buffered saline (PBS, pH 7.4) to a concentration of 1 mg/mL prior to use. The same batches of these venoms were used for each of the analyses to provide cross-experiment continuity. Four distinct equine F(ab’)_2_-based commercial antivenoms were used in this study (see Table 2 for full details), specifically: (i) the monospecific anti-*B. lanceolatus* antivenom BothroFav, (ii) two batches of the trispecific (including anti-*B. asper*) antivenom PoliVap-ICP, (iii) the polyspecific anti-*Bothrops* antivenom Soro antibotrópico-crotálico and, (iv) as a non-anti-*Bothrops* control antivenom, the polyspecific anti-*Crotalus* antivenom Soro anticrotálico. BothroFav was provided by MicroPharm Ltd., UK, while the other antivenoms were sourced from the CSRI antivenom collection previously donated to LSTM by Public Health England. The antibody concentrations of the antivenoms were determined by measuring the A280 nm using a Thermo Scientific Nanodrop Spectrophotometer, with each reading performed in triplicate. Naïve horse IgG (BIO-RAD) was used as a negative control antibody sample throughout this study.

### 4.2. Immunological Assays

#### 4.2.1. SDS-PAGE Gel Electrophoresis

As a precursor to Western blotting experiments, we used one dimensional SDS-PAGE gel electrophoresis to visualise the proteins present in the seven *Bothrops* venoms used in this study. Fifteen well 15% SDS-PAGE gels were hand cast using the following approach: (i) a resolving gel consisting of 3.75 mL H_2_O, 3.75 mL 40 % bis-acrylamide, 2.5 mL Tris pH 8.8 (1.5 M), 100 µL 10 % SDS, 60 µL 10 % ammonium persulfate (APS) and 7 µL N,N,N′,N′-Tetramethyl ethylenediamine (TEMED) and (ii) a stacking gel consisting of 2.5 mL H_2_O, 350 µL 40 % bis-acrylamide, 1 mL Tris pH 6.8 (1 M), 30 µL 10 % APS and 5 µL TEMED. Next, 10 µL of each venom (1 mg/mL) was mixed 1:1 (*v*/*v*) with reducing buffer (stock, 3.55 mL H_2_O, 2.50 mL glycerol, 1.25 mL pH 6.8 Tris (0.5 M), 2.0 mL 10 % SDS, 1.50 mL saturated Bromophenol blue, and with the addition of 150 µL β-mercaptoethanol per 850 µL reducing buffer prior to use) and subsequently heated for 3-5 min at 100 °C. After that, 10 µL of each sample was loaded on to the gel, alongside 5 µL of a molecular weight protein marker (Broad Range Molecular Marker, Promega, Southampton, UK), and the samples run at 200 V for 55 min using a Mini-PROTEAN Electrophoresis System (Bio-Rad, Watford, UK). Resulting gels were then stained at a final concentration of 0.1% (*w*/*v*) Coomassie blue R350 (0.4g of Coomassie blue R350 in 200 mL of 40% (*v*/*v*) methanol in H_2_O) and 10% (*v*/*v*) acetic acid overnight at room temperature (RT). Gels were then destained (50% H_2_O, 40% methanol, 10% glacial acetic acid) for at least 2 h at RT. Finally, a Gel Doc EZ Gel Documentation System (Bio-Rad) was used to visualise the resulting protein bands.

#### 4.2.2. Immunoblotting

For immunoblotting experiments of the venom proteins with the various antivenoms, SDS-PAGE gel electrophoresis was prepared as described above, but instead of Coomassie staining, proteins in the gels were transferred onto 0.2 µm nitrocellulose membranes using a Trans-Blot Turbo Transfer System following the manufacturer’s instructions (Bio-Rad). Following confirmation of protein transfer by reversible Ponceau S staining, the membranes were blocked in 5% non-fat dried milk (for blocking non-specific binding) in TBST buffer (0.01 M Tris-HCl, pH 8.5; 0.15 M NaCl; 1% Tween 20) and left overnight at 4 °C rocking at slow speed. Subsequently, blots were washed three times over 15 min with TBST before the addition of primary antibodies (either Soro anticrotálico, Soro antibotrópico-crotálico, BothroFav or PoliVap-ICP antivenom; all standardised to 50 mg/mL), which were diluted 1:5000 in 5% non-fat dried milk in TBST, and incubation for 2 h at RT. The immunoblots were then washed in triplicate with TBST as described above and incubated for two hours at RT with 50 mL of secondary antibodies, horseradish peroxidase (HRP)-conjugated Rabbit anti-horse IgG (Sigma, Gillingham, UK), diluted 1:2000 in PBS. Thereafter, the immunoblots were washed again with TBST and developed by the addition of DAB substrate (50 mg 3,3′-diaminobenzidine, 100 mL PBS and 0.024% hydrogen peroxide: Sigma) for 30 s, before washing with deionised water.

#### 4.2.3. Endpoint Titration Enzyme-Linked Immunosorbent Assay (ELISA)

Ninety-six well ELISA plates (Thermo Fisher Scientific, Winsford, UK) were coated with coating buffer (100 mM carbonate/Bicarbonate buffer, pH 9.6) containing 100 ng of each venom and incubated overnight at 4 °C. The plates were then washed three times with TBST before the addition of the blocking buffer, 100 µL of 5% non-fat milk in TBST, to each well, and further incubated at RT for two hours, followed by washing another three times with TBST. Next, 120 µL of primary antibodies (the same antivenoms as described for immunoblotting, alongside control normal horse IgG, all standardised to 50 mg/mL) were added to the plate in duplicate at an initial dilution of 1:100 in 5% non-fat milk in TBST, followed by five-fold serial dilutions across the plate and incubation at 4°C overnight. The plates were then washed again with TBST and incubated for two hours at RT with HRP-conjugated rabbit anti-horse IgG diluted at 1:1,000 in PBS. The plates were washed again before the addition of substrate (0.2% 2,2/-azino-bis (2-ethylbenzthiazoline-6-sulphonic acid) in citrate buffer (0.5 M, pH 4.0) containing 0.015% hydrogen peroxide (Sigma, UK)). Plates were gently shaken and incubated at RT for 15 min, before the signal was read spectrophotometrically at 405 nm on an LT-4500 microplate absorbance reader (Labtech, Heathfield, UK).

#### 4.2.4. Relative Avidity ELISA

Relative avidity ELISAs were performed in the same way as for the endpoint titration ELISAs, except that: (i) the same primary antibodies (i.e., antivenoms and normal horse control) were incubated at a single defined dilution (1:10,000) and (ii) after washing the primary antibody with TBST, wells were exposed to the chaotropic agent, ammonium thiocyanate (NH_4_SCN), at a range of concentrations (0–8 M) in duplicate for 15 min at RT. The plates were then washed with TBST, and all subsequent steps were the same as described for the endpoint titration ELISA. To permit direct and informative comparisons, reduction percentages (in terms of OD values) were calculated by subtracting 4 M NH_4_SCN readings from 0 M readings (the control).

### 4.3. Venom Activity Assays

#### 4.3.1. Metalloproteinase Activity Assay

To quantify snake venom metalloproteinase activity and inhibition of this activity by the various antivenoms under study, we used a previously described fluorescent kinetic enzymatic assay [29,40]. One microgram of each venom (1 mg/mL) and 6.91 µg (70 mg/mL) of the commercial antivenoms (Soro anticrotálico, Soro antibotrópico-crotálico, BothroFav and PoliVap-ICP) were first co-incubated in a water bath at 37 °C for 30 min. For determining baseline venom activity, we replaced antivenom with PBS, to act as venom only controls. A positive control venom with known metalloproteinase activity was also used for standardisation across all assays (1 µg; *Echis ocellatus*) [29], alongside a PBS (no venom) negative control. Post-incubation, 10 µL of each sample was added in triplicate to a 384-well Greiner microtitre plate. Thereafter, the quenched fluorogenic substrate ES010 (R&D Systems, Inc., Minneapolis, MN, USA) was prepared in reaction buffer (50 mM Tris-Cl pH 7.5, 150 mM NaCl) to a final substrate concentration of 10 µM, and 90 µL added to each well on the plate using a multichannel pipette. The assay was then read kinetically for 1 h at 25 °C using a FLUOstar Omega microplate reader (BMG Labtech, Aylesbury, UK) with an excitation wavelength of 320 nm and an emission wavelength of 405 nm. The enzymatic reaction was monitored by setting the gain adjustment on the instrument to 5% in wells where high activity was expected (e.g., positive control/venom only mixtures). Mean measures of fluorescence were then plotted against time to compare venom activity with baseline (negative controls) and positive control readings. For quantification, we calculated areas under the curves (AUCs) and the standard error of mean AUC readings for each sample in the 0-40 min interval; this time point was chosen as the time where all fluorescence curves had reached a plateau (maximum fluorescence). We then subtracted the mean of the relevant negative control readings from the venom readings and calculated the reduction percentage for all antivenom samples and re-plotted the triplicate readings with SEMs. Data analyses were performed using Prism v8 software (GraphPad, San Diego, CA, USA).

#### 4.3.2. Plasma Coagulation Assay

To quantify the coagulopathic activity of each of the venoms and the ability of the different antivenoms to inhibit this activity, we used a kinetic absorbance-based coagulation assay [28]. One microgram (100 ng/µL) of each venom and 3.75 µg (70 mg/mL) of the different commercial antivenoms were first co-incubated in a water bath at 37 °C for 30 min. The positive control used was 1 µg of *Echis ocellatus* venom based on its previously described potent procoagulant effect in this assay [29], and the negative control was plasma with the addition of PBS instead of venom. Citrated bovine plasma (Sterile Filtered, Biowest, Nuaille, France) was defrosted in a water bath, then centrifuged for 4 min at 805× *g* and precipitate discarded. Next, 10 µL of each venom-antivenom sample was added in triplicate to a 384-well Greiner plate, followed by the addition of 20 µL of 20 mM CaCl_2_ (prepared fresh for each assay), and then 20 µL of plasma, via use of a multidrop pipetting robot (Thermo Fisher Scientific, Winsford, UK). Data were then captured kinetically using a FLUOstar Omega microplate reader (BMG Labtech, Aylesbury, UK) and an optical density of 595 nm for 2 h at 25 °C. Mean measures of absorbance were then plotted against time to compare venom activity with baseline (negative control) and positive control readings. For quantification, we calculated the AUCs and the standard error of the mean AUCs using Prism v8 software (GraphPad, San Diego, CA, USA). We then subtracted the mean of the relevant negative control readings from the venom readings and calculated the reduction percentage of venom activity for all antivenom datasets with SEMs. Data analyses were performed using Prism v8 software (GraphPad, San Diego, CA, USA).

#### 4.3.3. Quantification of Fibrinogen Consumption

To explore the inhibitory capabilities of the antivenoms against the coagulopathic and fibrinogenolytic activity of the various *Bothrops* venoms, we used plasma spiking experiments followed by Clauss method-based quantification of fibrinogen consumption [41], as previously described [42]. Blood samples for the generation of human plasma were obtained according to Liverpool School of Tropical Medicine research tissue bank ethically approved protocols (REC ref. 11/H1002/9) from consenting healthy volunteers who confirmed they had not taken any anticoagulant treatments prior to blood collection for at least three months. Blood samples were collected in tubes containing acid citrate dextrose adenine (ACD-A) solution as anticoagulant, mixed gently and then centrifuged twice at 2500× *g* at 20–25 °C for 10 min to separate Platelet Poor Plasma (PPP).

Human PPP was spiked with either venom or venom and antivenom to assess the inhibitory capability of the commercial antivenoms against the depletion of fibrinogen. Twenty microlitres of human PPP was spiked with 0.6 ng of each *Bothrops* venom (or *E. ocellatus* venom as the positive control) or 0.9% saline as the negative control. All venom experiments were also repeated in the presence of each of the commercial antivenoms (0.5 µg), using a short preincubation step (37 °C for 5 min) prior to their addition to human PPP. Samples were then diluted 10-fold with imidazole buffer (pH 7.35), transferred to clean glass test tubes (10 × 75 mm^2^) and incubated at 37 °C for 120 s. Thereafter, 100 µL of 20 units/mL of thrombin reagent (Diagnostic Reagents Ltd, Thame, UK) was added and time measurements commenced. Tubes were gently tilted at regular intervals, returning to the water bath between tilting, and the time for the formation of a clot to occur recorded. All experiments were performed in duplicate.

## Figures and Tables

**Table 1:** The venoms used in this study.

Venom	Origin	Source
*B. lanceolatus*	Martinique	Latoxan, France
*B. caribbaeus*	St. Lucia	Donated by Kentucky Reptile Zoo, USA
*B. asper*	Costa Rica	CSRI historical collection
*B. jararaca*	Brazil	CSRI historical collection
*B. atrox*	Colombia	Kentucky Reptile Zoo, USA
*B. atrox*	Guyana	Kentucky Reptile Zoo, USA
*B. atrox*	Surinam	Kentucky Reptile Zoo, USA

**Table 2:** The commercial equine antivenoms used in this study.

Antivenom	Immunising Mixture	Lot #	Expiry Date	Antibody (mg/mL)	Manufacturer	
Soro anticrotálico	*C. d. cascavella* *C. d. collilineatus* *C. d. terrificus*	0304064/B	2006	40	Instituto Butantan, Brazil	
Soro antibotrópico-crotálico	*B. jararaca* *B. neuwiedi* *B. alternatus* *B. moojeni* *B. jararacussu* *C. durissus* *C. d. terrificus* *C. d. collilineatus*	1012308	2013	130	Instituto Butantan, Brazil	
BothroFav	*B. lanceolatus*	P4A561V	2020	92	MicroPharm Limited, UK	
PoliVap-ICP (2006)	*B. asper* *C. durissus* *L. muta*	3950406LO	2009	64	Instituto Clodomiro Picado, Costa Rica	
PoliVap-ICP (2013)	*B. asper* *C. durissus* *L. muta*	5270513POLQ	2016	70	Instituto Clodomiro Picado, Costa Rica	

## Data Availability

The data presented in this study are available in File S1.

## Supplementary Materials

The following supporting information can be downloaded at: https://www.mdpi.com/article/10.3390/toxins15010001/s1, Figure S1: The metalloproteinase activity of each venom and their inhibition by the commercial antivenoms as measured by kinetic fluorescent assay; Figure S2: The coagulopathic activity of each venom and their inhibition by the commercial antivenoms as measured by plasma coagulation assay; File S1: An multi-tabbed excel file containing the data presented in this study.
