# Supplementary material for: Commercial Antivenoms Exert Broad Paraspecific Immunological Binding and In Vitro Inhibition of Medically Important Bothrops Pit Viper Venoms
Source: Toxins (Basel). 2022 Dec 20;15(1):1. doi: 10.3390/toxins15010001 (PMC9862972; doi:10.3390/toxins15010001)
Supplement: Supplementary file 1 [file toxins-15-00001-s001.zip › toxins-2043404-supplementary.pdf]

<u>**Supplementary Information**</u>

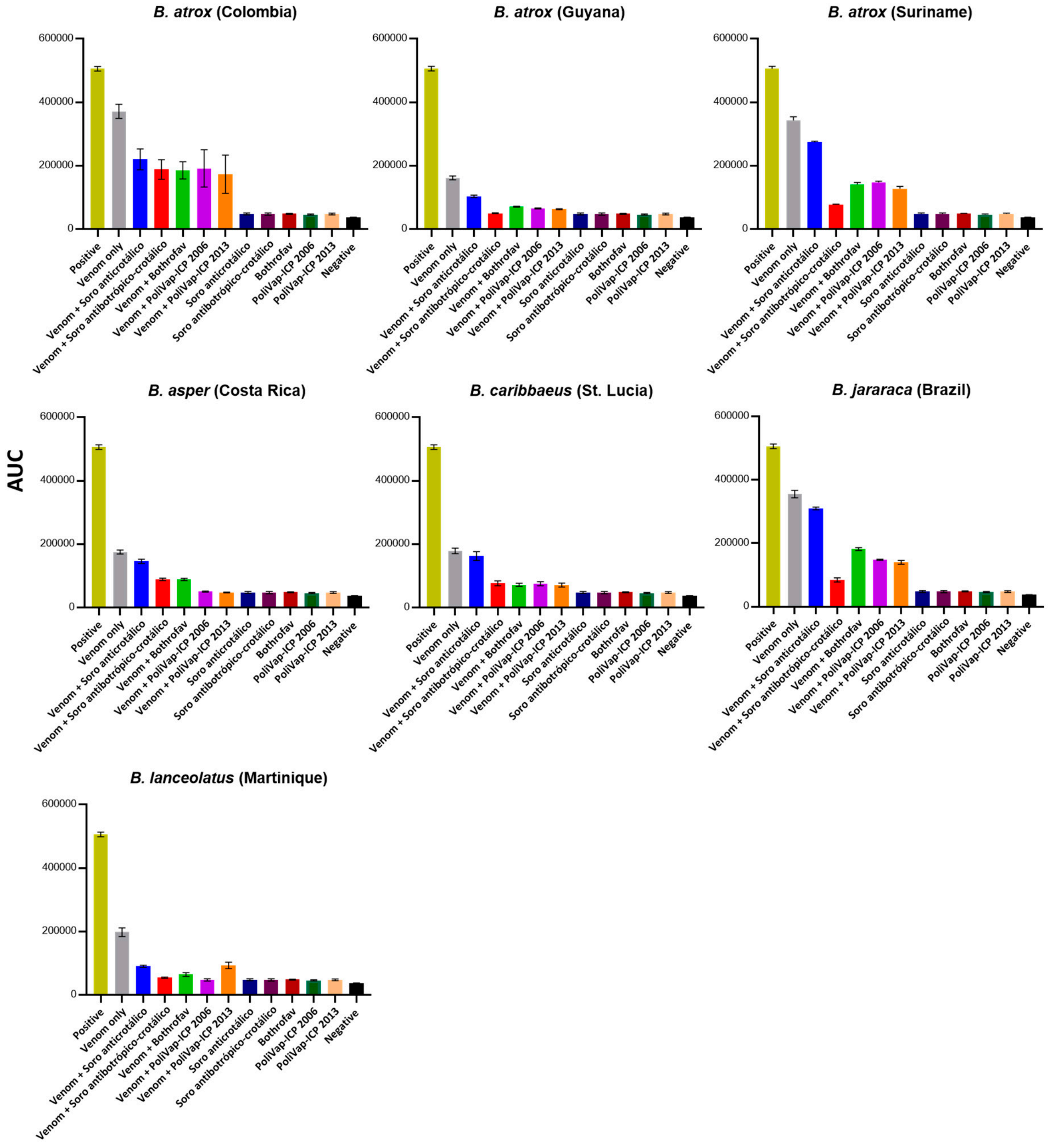

Figure S1. The metalloproteinase activity of each venom and their inhibition by the commercial antivenoms as measured by kinetic fluorescent assay. The SVMP venom activity (1 µg; 1mg/ml) of each venom is displayed as area under the kinetic curve (AUC) of fluorescence (320 nm excitation and 405 nm emission over 40 mins). The positive control used across all experiments was *Echis ocellatus* venom, while the negative control was PBS. The data displayed represents the mean AUC of triplicate measurements and error bars represent standard error of the mean (SEM).

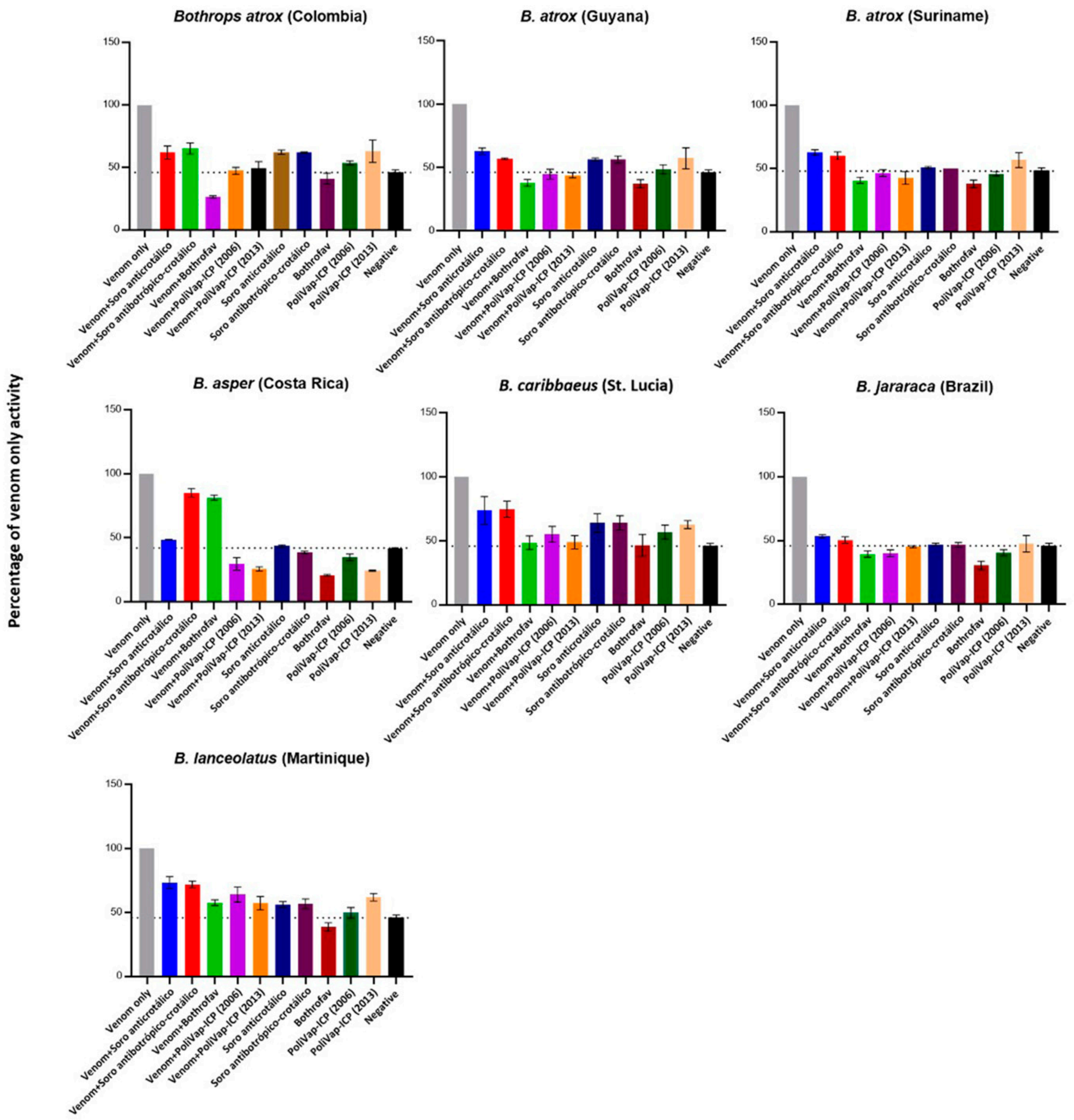

Figure S2. The coagulopathic activity of each venom and their inhibition by the commercial antivenoms as measured by plasma coagulation assay. The coagulopathic venom activity (1 µg; 100 ng/µl) of each venom displayed as area under the kinetic curve (AUC) of the percentage of plasma clotting (normalised to venom only readings), with error bars representing standard error of the mean (SEM). The dashed line represents normal clotting (i.e., negative control [PBS] readings).
